# How many births in sub-Saharan Africa and South Asia will not be attended by a skilled birth attendant between 2011 and 2015?

**DOI:** 10.1186/1471-2393-12-4

**Published:** 2012-01-17

**Authors:** Sonya Crowe, Martin Utley, Anthony Costello, Christina Pagel

**Affiliations:** 1 Clinical Operational Research Unit, University College London, London, UK; 2 Centre for International Health and Development, University College London Institute of Child Health, London, UK

## Abstract

**Background:**

The fifth Millennium Development Goal target for 90% of births in low and middle income countries to have a skilled birth attendant (SBA) by 2015 will not be met. In response to this, policy has focused on increasing SBA access. However, reducing maternal mortality also requires policies to prevent deaths among women giving birth unattended. We aimed to generate estimates of the absolute number of non-SBA births between 2011 and 2015 in South Asia and sub-Saharan Africa, given optimistic assumptions of future trends in SBA attendance. These estimates could be used by decision makers to inform the extent to which reductions in maternal mortality will depend on policies aimed specifically at those women giving birth unattended.

**Methods:**

For each country within South Asia and sub-Saharan Africa we estimated recent trends in SBA attendance and used these as the basis for three increasingly optimistic projections for future changes in SBA attendance. For each country we obtained estimates for the current SBA attendance in rural and urban settings and forecasts for the number of births and changes in rural/urban population over 2011-2015. Based on these, we calculated estimates for the number of non-SBA births for 2011-2015 under a variety of scenarios.

**Results:**

Conservative estimates are that there will be between 130 and 180 million non-SBA births in South Asia and sub-Saharan Africa from 2011 to 2015 (90% of these in rural areas). Currently, there are more non-SBA births per year in South Asia than sub-Saharan Africa, but our projections suggest that the regions will have approximately the same number of non-SBA births by 2015. We also present results for each of the six countries currently accounting for more than 50% of global maternal deaths.

**Conclusions:**

Over the next five years, many millions of women within South Asia and sub-Saharan Africa will give birth without an SBA. Efforts to improve access to skilled attendance should be accompanied by interventions to improve the safety of non-attended deliveries.

## Background

Among the eight United Nations Millennium Development Goals (MDGs), progress towards the fifth, to reduce maternal mortality by three quarters between 1990 and 2015, has been particularly slow [1-4]. Although a recent global review [5] suggests levels of maternal mortality in developing regions have fallen since 1990, it has become increasingly clear that significant improvements in health care for women are required if the goal is to be achieved. This is particularly the case for sub-Saharan Africa and South Asia, in which at least 87% of the estimated annual 342,900 maternal deaths worldwide occur according to recent estimates, with over 50% of all maternal deaths occurring in only six countries (India, Nigeria, Pakistan, Afghanistan, Ethiopia and the Democratic Republic of Congo).

Increasing the availability of skilled health professionals to supervise deliveries has been identified as a key strategy for reducing maternal deaths, with the proportion of births attended by a skilled birth attendant (SBA) adopted as a direct indicator for MDG5 [6,7]. The global target is for at least 90% of births to be supervised by a SBA worldwide by 2015 [8]. Overall, the proportion of SBA-births in developing regions increased from 53% in 1990 to 61% in 2007, yet in South Asia and sub-Saharan Africa this figure remained less than 50% [9]. It is also of note that the proportion of SBA births in these regions is significantly lower in rural than in urban areas [10,11].

Although it has proved difficult to establish a causal link between SBA births and maternal mortality rates [12], estimates suggest that SBA presence at delivery could prevent around 16% to 33% of maternal deaths [13]. In addition, SBA attendance at birth may impact on rates of stillbirths and neonatal mortality related to intrapartum events. To this end, local and international effort focuses on improving access to SBA attendance at birth throughout the developing world, including activities such as training attendants, increasing access to health facilities and allocating health resources more equitably among rural and urban areas [14].

Despite concerted effort to increase SBA attendance, in the short to medium term many women in the developing world will continue to give birth without the supervision of a SBA. Thus, alongside striving for progress in improving access to SBAs, it has been argued that measures must be targeted at those women who will not be covered by these interventions [15-18]. The aim of this work is to inform policy-makers and those planning health services as to the scale of unattended births that might be expected over the coming years, both for individual countries and at regional level.

In this paper we use data from existing sources to estimate the number of non-SBA births that will take place between 2011 and 2015 in sub-Saharan Africa and South Asia, both in rural and urban areas. Due to large variation in the level of skilled birth attendance between countries, the analysis is performed for each country individually and subsequently aggregated to form the regional estimates presented in the main body of text; individual country level estimates are given in additional file 1: country-level results. We consider six scenarios defined by assumptions regarding the annual increase in the proportion of SBA births (based on current trends and increasingly optimistic projections) and the projected number of births (high and low fertility estimates).

We note that our work is intended to raise key policy issues rather than act as an accurate forecasting model.

## Methods

For any given country, future SBA attendance will be the product of policy initiatives, migration patterns and other demographic changes, with the first in particular being subject to potentially sudden changes. For example, in Malawi, births attended by traditional birth attendants were made illegal in 2009, with a stepwise increase in institutional delivery, although the government has since rescinded this law. For these reasons, we have not attempted to generate precise predictions for the number of non-SBA attended births using sophisticated statistical techniques based on historical data. Rather, our aim was to gauge, broadly, the scale of the number of non-SBA attended births under a range of assumptions about future SBA trends.

When we have, necessarily, made simplifying modelling assumptions, we have tended to be optimistic about future trends in SBA attendance, so that our projections represent optimistically low estimates for the number of women giving birth without a skilled birth attendant. The deliberate presentation of optimistically low estimates is intended to highlight the scale of unattended births that are essentially inevitable under plausible future projections. For example, when considering which methodology to adopt for estimating trends in SBA attendance we felt it inappropriate and infeasible to determine bespoke fits for each country based on the observed historic data due to its sparseness and the policy-driven nature of the trends over recent years. Instead, we used the simple approach outlined below.

**Figure 1:**
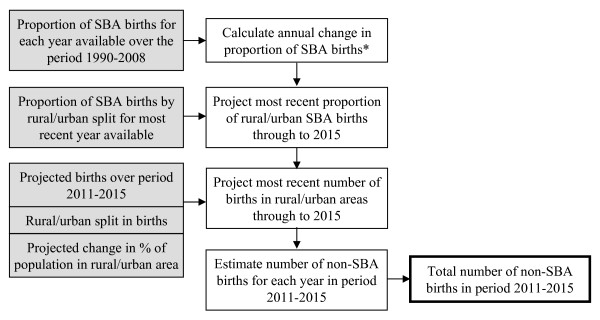
**Methodological steps**. The methodological steps performed in the analysis for each country and each fertility assumption. Data inputs are indicated with shaded boxes, while outputs are indicated with a bold border. *Note that we additionally considered more optimistic projections for trends in SBA attendance from the end of 2010 onwards, based on scaling multiples of recent trends; see text for more details.

The basis of our analysis can be followed using the flow diagram set out in Figure 1; the mathematical equations and exact procedures used are detailed in additional file 2: mathematical notation and analysis. We give a brief description of the methods below. The same method was applied to each of the 8 and 47 nations comprising the South Asia and sub-Saharan Africa regions respectively (as specified by the country groups set out by the World Bank [19]). For a detailed description of how the parameters were estimated and how we surmounted problems with data availability for individual countries, see additional file 2: mathematical notation and analysis.

### Step 1: Estimating the recent trend in SBA attendance for each country

The starting point for estimating the recent trend was the proportion of all births attended by a SBA for each year over the period 1990-2008 for which data was available. Due to the policy-driven nature of SBA rates over this period, an underlying natural trend to which these data can be fitted does not necessarily exist. Additionally, attempting to fit a complex model would be inappropriate for the large number of countries with 3 or fewer data points available (63% of countries). Instead, we calculated the annual percentage change between each pair of available data points and took an average since 2000 to estimate the recent year-on-year trend in SBA attendance. Note that we were unable to disaggregate SBA trends by rural/urban area as Demographic Health Surveys (DHS) data on differential SBA attendance at two time points was available for only fifteen countries. To explore the impact on our results of using aggregated SBA trends, we estimated rural- and urban-specific trends for those countries where the data were available and compared estimates of future non-SBA deliveries using these non-aggregated trends with estimates generated using aggregated trends.

### Step 2: Projecting SBA attendance up to 2010

We used the estimates of recent year-on-year trend in SBA attendance from step 1 to project forward the SBA attendance for rural and urban areas to the end of 2010 for all countries from the most recent available data for SBA attendance for rural and urban areas, thus giving a common starting point for future projections.

### Step 3: Projecting SBA attendance between 2011 and 2015

The proportion of births attended by a SBA in rural and urban areas for each year from 2011 to 2015 was estimated from the 2010 estimates (above), assuming the annual change in SBA coverage calculated at step 1 is carried forward from 2011 to 2015. We also considered scenarios with more optimistic and very optimistic projected annual changes in SBA coverage (see below for details).

### Step 4: Estimating the number of births in rural and urban areas between 2011 and 2015

We estimated the total number of births in rural and urban areas each year by using the most recently observed rural/urban split in number of births and projections for the total number of births from 2011-2015. We considered both a low and a high fertility scenario for birth projections (see below for details). In order to account for migration patterns, we assumed that the relative proportion of rural and urban births changes over time by the same amount as the projected change in the relative proportion of the population in rural and urban areas (see additional file 2: mathematical notation and analysis for details).

### Step 5: Projecting the number of births not attended by SBAs between 2011 and 2015

Finally, we estimated the number of non-SBA births in rural and urban areas in each year from 2011 using the above projections for SBA coverage and for the number of births each year in each area.

The country-specific estimates were aggregated to give a projected number of non-SBA births in sub-Saharan Africa and South Asia annually over the period 2011-2015 (in rural and urban areas), and the total between 2011 and 2015 inclusive.

The analysis was conducted for each of six scenarios defined by assumptions regarding the annual increase in the proportion of SBA births and assumptions for the projected number of births, as set out in Table 1. To incorporate plausible scenarios for the potential impact of improving access to skilled birth attendance in the future, the analysis in steps 3 onwards was also performed using *double *(more optimistic) and *quadruple *(very optimistic) the annual percentage change in the proportion of SBA births observed since 2000. To ensure that these more optimistic annual increases remained within the limits of plausibility, we capped any individual country's annual increase at the maximum observed recent annual trend in SBA attendance across all countries (Bhutan, 17% annual improvement). For the twelve countries where the observed recent annual trend was negative, we assumed a constant SBA attendance over time (i.e. no further decrease) in all six scenarios. We used two sets of UN projections reflecting high and low fertility assumptions (see data sources section) for the number of projected births.

**Table 1:** Analysis scenarios

	Annual% change in SBA delivery based on current trend	More optimistic projection of annual% change in SBA delivery *	Very optimistic projection of annual% change in SBA delivery ^†^	High fertility assumption for projected births	Low fertility assumption for projected births
Scenario 1	X			X	

Scenario 2		X		X	

Scenario 3			X	X	

Scenario 4	X				X

Scenario 5		X			X

Scenario 6			X		X

The six scenarios used in the analysis, each of which assumes one of three projections in changes in SBA attendance and one of two fertility assumptions regarding projected number of births. *Annual percentage change is double (^†^quadruple) the current trend, but for any given country, this value is capped at the maximum observed current trend across all countries (Bhutan, 17% annual improvement). A lower bound of zero was applied to all scenarios.

## Data sources

The proportion of SBA births for all areas (i.e. urban and rural jointly) for each country within sub-Saharan Africa and South Asia were obtained from the UN website for MDG indicators [20] over the period 1990 - 2008. If there was a more recent value given in the UNICEF http://childinfo.org report [21] for a country, then this was added as an extra observation. Most of this summary data comes from individual country DHSs, which use a standardised methodology and sampling framework and have strict criteria on data quality, thereby allowing comparison between countries and over time. Across DHSs the definition of an SBA can vary, in particular regarding whether or not traditional birth attendants (TBAs) are included (for instance TBAs are included for DR Congo, but excluded for Bangladesh). Rather than attempt to harmonise these definitions, we note that the inclusion of TBAs by some countries in their skilled birth attendance estimates make our estimates for the number of women giving birth without skilled attendance in those countries optimistic, according to the WHO definition of skilled birth attendance.

The most recent proportion of SBA births for rural and urban areas was obtained from the UNICEF http://childinfo.org website [21], which lists the most recently available value for these indicators for each country.

Estimates for the future annual number of births for the individual countries within sub-Saharan Africa and South Asia were taken from projections by the Population Division of the Department of Economic and Social Affairs of the United Nations Secretariat [22] for the period 2010-2015. Two of the possible projection scenarios set out by the UN Population Division were adopted; a low fertility and a high fertility assumption [23]. These were chosen to provide lower and upper estimates. The rural/urban split of projected births was estimated using the proportion of births recorded in the most recent individual country DHS [24] as rural and projections for the average annual change in the proportion of the population in rural and urban areas by the Population Division of the Department of Economic and Social Affairs of the United Nations Secretariat [22] for the period 2005-2015.

## Results

No data were available for the proportion of SBA births for Senegal, Mayotte, Seychelles and Tanzania, hence these countries are not included in our analysis. It was not possible to calculate an annual change in SBA attendance for Congo, so we adopted the average rate of change over the region.

Additionally, the most recent proportions of SBA births for rural and urban areas were not available for Chad, Maldives, Mauritius and Sudan. For these countries we used the average rural/urban split over the region as described in additional file 2: mathematical notation and analysis.

The rural/urban split of projected birth numbers was not available for Afghanistan, Bhutan, Maldives, Sri Lanka, Angola, Gambia, Guinea-Bissau, Mauritius, Sao Tome and Principe, and Somalia. Rural/urban splits in the number of births for these countries were estimated from regional averages as described in additional file 2: mathematical notation and analysis.

In the 15 countries where data were available to perform the analysis using rural- and urban-specific trends in SBA coverage, our predictions were not markedly different from those obtained using the aggregated trend. We note that across these 15 countries there was no consistent pattern in rural versus urban trends in SBA coverage that could have been applied to the remaining nations.

**Figure 2:**
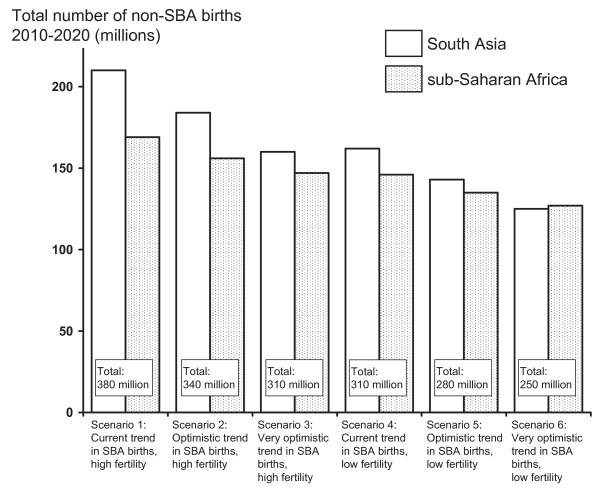
**Non-SBA birth projections**. Projections for the estimated number of non-SBA births in South Asia and sub-Saharan Africa during the period 2011-2015 inclusive for the six scenarios set out in Table 1. Total non-SBA births over both regions are given to the nearest 5 million. In all scenarios, at least 90% (85%) of estimated non-SBA births were in rural areas for South Asia (sub-Saharan Africa). Note that we were not able to calculate estimates for Senegal, Mayotte, Seychelles and Tanzania.

**Figure 3:**
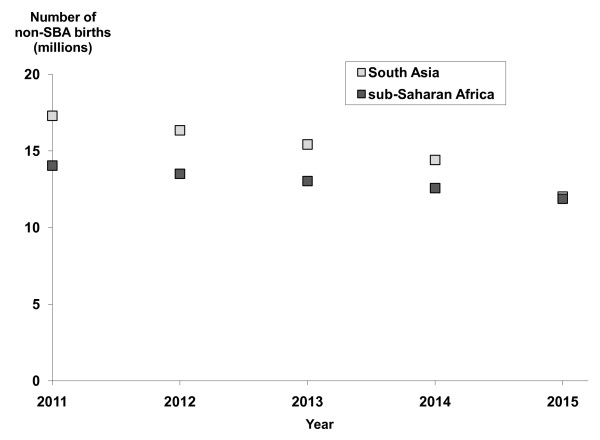
**Non-SBA projections over time**. Estimated total annual number of non-SBA attended births over the period 2011-2015 (inclusive) for scenario 5: optimistic trend in SBA births, low fertility.

Projections for the estimated number of non-SBA births in South Asia and sub-Saharan Africa during the period 2011-2015 are shown in Figure 2 for the six scenarios defined in Table 1. The total estimated number of non-SBA births ranges from 130 to 180 million (consistent with current estimates from Prata [18]). Note that in all scenarios at least 90% (85%) of non-SBA births in South Asia (sub-Saharan Africa) were projected to take place in rural areas. To illustrate the change in non-SBA births between 2011 and 2015, the annual numbers of non-SBA births for a single scenario (scenario 5) are shown in Figure 3.

Projections for the estimated number of non-SBA births in India, Nigeria, Pakistan, Afghanistan, Ethiopia, and the Democratic Republic of the Congo during the period 2011-2015 inclusive are given in Table 2 for the six scenarios defined in Table 1 in the order that they contribute to global maternal deaths. In 2008, these six countries accounted for over 50% of the world's maternal deaths [5].

**Table 2:** Results for the six countries with highest annual number of maternal deaths

Country	Current annual rate of improvement in SBA attendance	Estimated 2010 SBA attendance (%)		Estimated non-SBA births 2011-2015 (millions) for each scenario					
		**Urban areas**	**Rural areas**	**1**	**2**	**3**	**4**	**5**	**6**

India	1.4%	78	40	69	66	60	55	53	48

Nigeria	-0.7%	66	27	19	19	19	17	17	17

Pakistan	10.1%	80	40	10	7	7	8	6	6

Afghanistan	4.9%	49	10	6	6	5	5	5	5

Ethiopia	0.4%	45	3	16	16	16	14	14	14

Democratic Republic of the Congo	3.4%	100	70	2	1	1	2	1	1

The current annual trend in SBA attendance and the estimated SBA attendance in 2010 in urban and rural areas are listed for India, Nigeria, Pakistan, Afghanistan, Ethiopia, and the Democratic Republic of the Congo. Projections for the estimated number of non-SBA births during the period 2011-2015 (inclusive) for the six scenarios set out in Table 1 are also given.

## Discussion

### Key findings

By using existing data and projections on future population trends and by considering optimistic scenarios for improvements in SBA attendance, we generated estimates of the total number of deliveries that would not have a skilled attendant over the coming decade in sub-Saharan Africa and South Asia, given optimistic assumptions of future trends in SBA attendance. These estimates were generated with the intention of raising key policy issues rather than providing accurate forecasts. Our estimates varied from 130 million unattended deliveries over the next five years in the best case scenario to 180 million deliveries in the worst case, representing 42% to 48% of the estimated number of births in these two regions. By far the majority of non-SBA births will occur in rural areas; whilst roughly 65% (60%) of the population will be rural over the next five years in South Asia (sub-Saharan Africa), our results suggest that at least 90% (85%) of non-SBA births will occur in rural areas.

Given the inherent limitations of country-level data over two diverse regions, and the fact that future policy in each country is unknown, we believe that attempting precise predictions of future non-SBA attendance would be inappropriate. Our intent was not to estimate the impact of policies aimed at increasing SBA attendance, but rather to estimate the extent to which targets for reducing maternal mortality, stillbirths and neonatal mortality related to intrapartum events, will depend not only on improving SBA attendance but also on preventing these adverse outcomes in unattended births. Where we have made modelling assumptions, we took an optimistic view of the future trends in SBA attendance.

Although currently South Asia has lower SBA rates than sub-Saharan Africa, recent trends suggest that South Asia is increasing access to skilled birth attendance more rapidly than sub-Saharan Africa. This, in combination with UN population projections, results in a prediction that by 2015 there will be approximately the same number of unattended births in sub-Saharan Africa as in South Asia.

### Limitations

In our analysis we assume a constant rate of change in the proportion of SBA births over time, based on recent observed changes at a country level. We acknowledge that this assumption is somewhat unrealistic, particularly at higher rates of SBA attendance, where ceiling effects come into play. However, we felt that, given the inappropriateness of more complex models, this approach was fit for purpose. To avoid using entirely unrealistic annual trends, we capped projected trends at the maximum observed recent value across all countries (Bhutan, 17% annual improvement).

It is assumed within the analysis that the relative proportion of rural and urban births changes over time by the same amount as the projected change in the relative proportion of the population in rural and urban areas, which is not necessarily the case as the demography and birth pattern of migrants is unclear. It is also not clear that new migrants to a city will follow the pattern of SBA attendance in urban areas, particularly as non-SBA deliveries can be culturally motivated. We note that if we were to assume instead that birth rates among migrants to urban areas drop, then our estimate of the relative proportion of rural births would be higher.

For countries where time-series data were not available regarding the proportion of SBA births, the most recent proportions of SBA births for rural and urban areas or the rural/urban split of projected birth numbers, regional averages were adopted. However, we note that our regional estimates are not particularly sensitive to different ways of assigning values to countries with missing data, especially given the relatively small number of countries affected.

Finally, we note that definitions of what qualifies as skilled attendance at birth have been variable over time and by region, which will have affected calculated trends. We have tried to mitigate this problem as far as possible by using UN figures throughout, although we acknowledge that across DHSs definitions can vary, particular regarding whether or not traditional birth attendants (TBAs) are included. We note that the inclusion of TBAs by some countries in their data (for instance Democratic Republic of Congo) will make our estimates for the number of non-SBA births, as defined by the WHO, too low.

### Policy implications

Currently in rural areas, births without a skilled birth attendant and without access to life-saving drugs are the commonest practice for millions of mothers in the poorest countries where mortality rates are highest. Our projections suggest that even with very optimistic increases in SBA attendance, at least a hundred million women will give birth without an SBA over the next five years, the overwhelming majority in rural areas. For policymakers this presents two major challenges: how to accelerate progress towards increasing accessibility to SBAs and institutional deliveries in more remote areas, and how to reduce the risks associated with unattended home deliveries. New efforts and strategies are needed to accelerate the rate of progress in increasing SBA coverage and to evaluate alternative interventions to reduce maternal deaths, stillbirths and neonatal deaths related to intrapartum events in unattended births [18]. For example, efforts to increase SBA coverage may include training traditional birth attendants [17,18] or greater national support for nurses, midwives and doctors [25]. Interventions to reduce adverse outcomes in births unattended by an SBA may include, for example, provision of oral uterotonics [18,26] and/or clean delivery kits [27] to mothers or postnatal home visits [28] to identify problems. We have previously attempted to model the impact of outreach strategies to increase access to life-saving drugs such as misoprostol for haemorrhage and antibiotics for treatment of sepsis in the post-partum period [16].

There exists large variation between countries in SBA coverage, trends in coverage (additional file 1: country-level results) and maternal mortality. At the same time, we recognise that policy initiatives to increase the numbers of women who can access a skilled birth attendant are insufficient if the quality and user-friendliness of services remains poor. There can be huge differences in the service provided by an SBA across countries and between rural and urban areas. Many of the deliveries reported as attended by an SBA are in countries where SBAs are not guaranteed to have access to supervision, regular refresher training, a regular supply of essential drugs, or easy access to referral services. These issues need to be addressed and improved concurrently with improvements in SBA attendance rates. We believe that our projections of non-SBA attended births, in conjunction with recent mortality estimates^5^, can usefully inform policy decisions aimed at reducing maternal deaths, still births and neonatal deaths at a country level.

## Conclusions

Over the next five years, at least a hundred million women within South Asia and sub-Saharan Africa will continue to give birth without a skilled birth attendant (SBA), even under highly optimistic projections for improvements in SBA coverage over this period. Women giving birth without an SBA are overwhelmingly in rural areas where it is intrinsically harder to increase access to medically trained personnel. Thus alongside a continued drive to improve SBA attendance, policies aimed specifically at those women giving birth unattended are needed in order to reduce maternal mortality.

## Competing interests

This work was supported with discretionary funds from the Clinical Operational Research Unit at University College London. We declare that we have no conflicts of interest.

## Authors' contributions

AC had the original idea for the study. SC and ACP designed and performed the analysis, in discussion with MU. SC wrote the first draft of the paper and all authors have read, commented on, contributed to and approved the final manuscript.

## Pre-publication history

The pre-publication history for this paper can be accessed here:

http://www.biomedcentral.com/1471-2393/12/4/prepub

## Supplementary Material

Additional file 1 **Country-level results**. This file contains, for each country, the most current proportion of SBA births available (with corresponding year), the estimated proportion of SBA births for 2010 in urban and rural areas and the projected number of total non-SBA births in the period 2011-2015 (inclusive) for scenarios 1-6. Also listed for each country is the range (from scenario 1 to scenario 6) in the estimated proportion of SBA births for 2015 in urban and rural areas. Click here for file

Additional file 2 **Mathematical notation and analysis**. This file contains the mathematical notation and analysis used in deriving the estimated number of births between 2011 and 2015 *for each country*. Click here for file
