# Supplementary material for: How many births in sub-Saharan Africa and South Asia will not be attended by a skilled birth attendant between 2011 and 2015?
Source: BMC Pregnancy Childbirth. 2012 Jan 17;12:4. doi: 10.1186/1471-2393-12-4 (PMC3274439; doi:10.1186/1471-2393-12-4)
Supplement: Additional file 1 — Country-level results. This file contains, for each country, the most current proportion of SBA births available (with corresponding year), the estimated proportion of SBA births for 2010 in urban and rural areas and the projected number of total non-SBA births in the period 2011-2015 (inclusive) for scenarios 1-6. Also listed for each country is the range (from scenario 1 to scenario 6) in the estimated proportion of SBA births for 2015 in urban and rural areas. [file 1471-2393-12-4-S1.DOC]

Additional file 1: Country-level results

We list, for each country, the most current proportion of SBA births available (with corresponding year), the estimated proportion of SBA births for 2010 in urban and rural areas and the projected number of total non-SBA births in the period 2011-2015 (inclusive) for scenarios 1-6. Also listed for each country is the range (from scenario 1 to scenario 6) in the estimated proportion of SBA births for 2015 in urban and rural areas.
