# Supplementary material for: How many births in sub-Saharan Africa and South Asia will not be attended by a skilled birth attendant between 2011 and 2015?
Source: BMC Pregnancy Childbirth. 2012 Jan 17;12:4. doi: 10.1186/1471-2393-12-4 (PMC3274439; doi:10.1186/1471-2393-12-4)
Supplement: Additional file 2 — Mathematical notation and analysis. This file contains the mathematical notation and analysis used in deriving the estimated number of births between 2011 and 2015 for each country. [file 1471-2393-12-4-S2.DOC]

Additional file 2: Mathematical notation and analysis

In this appendix we set out the mathematical notation and analysis used in deriving the estimated number of births between 2011 and 2015 *for each country*.

**Notation**

Let $y_{k}$be the ordered years for which data on SBA coverage is available, where $1990\leq y_{k}\leq2008$

Let $c_{k}$be the annual change in SBA coverage between years $y_{k}$ and $y_{k+1}$

Let $z$ be the year for which most recent data on SBA coverage is available, $z\leq2008$.

Let $t$ be the year for which most recent data on the proportion of births in rural areas is available, $t\leq2008$.

Let $c$ be the mean annual percentage change in skilled birth attendance in the period 2000 to $z$.

Let *n* be an integer value used for projecting improvements in future SBA coverage.

Let $d_{a,j}$, $d_{r,j}$ and $d_{u,j}$ denote the proportion of deliveries attended by a skilled birth attendant in all, rural and urban areas respectively in the year *j*.

Let $X_{j}$ be the number of projected births in the year *j*.

Let $r_{j}$ denote the proportion of births in rural areas in the year *j*. Then $1-r_{j}$is the proportion of births in urban areas in the year *j*.

Let $p$ denote the average projected annual rate of change of the proportion of the population in rural areas over the period 2005-2015.

Let $B_{r,j}$ and $B_{u,j}$ denote the number of births *without* skilled birth attendance in rural and urban areas respectively in year *j*.

**Analysis**

*Estimating the trend in SBA attendance for each country*

Using the proportion of all births attended by a SBA for each year over the period 1990-2008 for which data was available, we calculated the annual percentage change in the proportion of SBA births between each pair of available data points:

$c_{k}=\left(\frac{d_{a,y_{k+1}}}{d_{a,y_{k}}}\right)^{\frac{1}{y_{k+1}-y_{k}}}-1$ (1)

Each year *j* between $y_{1}$ and $z$ is allocated $c_{k}$ such that $y_{k}<j\leq y_{k+1}$. The recent trend, *c*, was then calculated as the mean of the $c_{k}$ between 2000 and *z*. For countries where no data was available post-2000, the most recent available annual percentage change prior to that was used.

*Estimating SBA attendance between 2011 and 2015*

Using this annual change, we extrapolated forward from the most recent recorded SBA proportion to estimate SBA coverage in 2010 (capped at 100%).

$d_{r,2010}=Min\left(1,d_{r,z}(1+c)^{2010-z}\right)$ (2)

$d_{u,2010}=Min\left(1,d_{u,z}(1+c)^{2010-z}\right)$ (3)

We then extrapolated forward from 2010 to estimate SBA coverage for each year from 2011 to 2015 using a multiple, *n*, of the annual change, which depends on the scenario. The proportion of SBA births in year *j* for $2011\leq j\leq2015$ in rural and urban areas is estimated as:

$$d_{r,j}=Min\left(1,d_{r,2010}(1+nc)^{j-2010}\right)$$

$d_{u,j}=Min\left(1,d_{u,2010}(1+nc)^{j-2010}\right)$,

where $n=1$ for scenarios 1 and 4, $n=2$ for scenarios 2 and 5 (more optimistic projections) and $n=4$ for scenarios 3 and 6 (very optimistic projections) (see Table 1 in main body of paper).

Note that we assume that the annual percentage change, *c*, in SBA coverage is the same for rural and urban regions. In the optimistic scenarios, we cap any individual country's annual increase at the maximum observed recent annual trend in SBA attendance across all countries (Bhutan, 17% annual improvement). For countries where the most recent value was not available by rural/urban split, the average regional ratio for rural/urban split was normalised to the country’s most recent proportion of SBA births for all areas. Note that for countries with a negative trend in skilled birth attendance (*c*<0), we set the annual change to zero. All proportions of SBA births are capped at 100%.

*Estimating the number of births not attended by SBAs between 2011 and 2015*

The proportion of births in rural areas in year *j* for $2011\leq j\leq2015$ is estimated as:

$r_{j}=r_{t}\left(1+p\right)^{j-t}$ (4)

The number of births in rural and urban areas without skilled birth attendance in year *j* for $2011\leq j\leq2015$ is estimated as:

$B_{r,j}=X_{j}r_{j}\left(1-d_{r,j}\right)$ (5)

$B_{u,j}=X_{j}(1-r_{j})\left(1-d_{u,j}\right)$ (6)

Note that the regional average split between rural and urban births was adopted for countries where the relative proportions of rural and urban births were unavailable. Note also that we assume that the relative proportion of rural and urban births changes over time by the same amount as the projected change in the relative proportion of the population in rural and urban areas.

*Regional totals*

For each region the total number of births without skilled birth attendance was calculated by summing the number of births for each country.
